# Atezolizumab Monotherapy or Plus Chemotherapy in First-Line Treatment for Advanced Non-Small Cell Lung Cancer Patients: A Meta-Analysis

**DOI:** 10.3389/fimmu.2021.666909

**Published:** 2021-06-02

**Authors:** Dan-Ni Li, Wen-Qing Lu, Bo-Wen Yang, Ling-Yun Zhang, Bo Jin, Shuo Wang, Xiao-Fang Che, Ce Li, Yun-Peng Liu, Xiu-Juan Qu

**Affiliations:** ^1^ Department of Medical Oncology, The First Hospital of China Medical University, Shenyang, China; ^2^ Key Laboratory of Anticancer Drugs and Biotherapy of Liaoning Province, The First Hospital of China Medical University, Shenyang, China; ^3^ Liaoning Province Clinical Research Center for Cancer, Shenyang, China

**Keywords:** non-small cell lung cancer (NSCLC), programmed cell death-ligand 1 (PD-L1), atezolizumab, chemotherapy, first-line

## Abstract

**Background:**

Atezolizumab plus chemotherapy has been recommended as a first-line treatment option for patients with advanced non-small cell lung carcinoma (NSCLC) irrespective of programmed cell death-ligand 1 (PD-L1) expression. Currently, little is known about the efficacy and treatment-related adverse effects (TRAEs) of subtracting chemotherapy from the combination for patients with high PD-L1 expression. Thus, we performed an indirect comparison between atezolizumab plus chemotherapy and atezolizumab alone.

**Methods:**

A total of five eligible randomized controlled trials (RCTs) were identified from PubMed, EMBASE, and Cochrane Central controlled trial registries, using keywords including atezolizumab, PD-1, PD-L1, NSCLC, and RCT. The clinical outcomes of objective response rate (ORR), progression-free survival (PFS), OS, and TRAEs were extracted and evaluated. Using indirect analysis, the efficacy and TRAEs were compared between arm A (atezolizumab plus chemotherapy) and arm C (atezolizumab), linked by arm B (chemotherapy).

**Results:**

Direct comparison revealed that both atezolizumab plus chemotherapy (HR 0.65, *P* = 0.003) and atezolizumab alone (HR 0.59, *P* = 0.010) significantly improved OS compared with chemotherapy. More importantly, the indirect comparison showed that atezolizumab plus chemotherapy was not superior to atezolizumab regarding OS (RR 1.10, *P* =0.695) and ORR (RR 1.11, *P* = 0.645). However, patients who received atezolizumab combined with chemotherapy experienced more ≥ grade 3 TRAEs (RR 4.23, *P*<0.001) and TRAEs leading to drug discontinuation (RR 3.60, *P*<0.001) than those treated with atezolizumab monotherapy.

**Conclusions:**

Atezolizumab monotherapy might be a better treatment option for patients with advanced NSCLC and high PD-L1 expression than atezolizumab plus chemotherapy.

## Introduction

Lung cancer is the leading cause of cancer-related mortality (1). In recent years, immune checkpoint inhibitors, whether as a monotherapy or in combination with chemotherapy, have become the standard of care for first-line treatment of patients with advanced non-small cell lung cancer (NSCLC) that lack targetable driver mutations. Currently, pembrolizumab monotherapy, pembrolizumab plus chemotherapy, or atezolizumab monotherapy are the preferred options for patients with high programmed cell death ligand 1 (PD-L1) expression (2–4). Previous studies showed that for those with a PD-L1 tumor proportion score (TPS) of at least 50%, the addition of chemotherapy to pembrolizumab significantly improved objective response rate (ORR) and progression-free survival (PFS), though at the cost of increased treatment-related adverse effects (TRAEs). Overall survival (OS) was not different between these two treatment options (3). However, whether this conclusion is similar to atezolizumab, an anti-PD-L1 antibody, remains unknown.

With the IMpower150 study, atezolizumab plus bevacizumab, carboplatin, and paclitaxel has been approved by the Food and Drug Administration (FDA) and the European Medicines Agency (EMA) as a first-line treatment for patients with advanced non-squamous NSCLC, regardless of PD-L1 expression level (5). Similarly, atezolizumab in combination with nab-paclitaxel and carboplatin also showed a significant and meaningful improvement in OS compared with chemotherapy in the same population (6). For patients with advanced squamous cell lung cancer, atezolizumab plus nab-paclitaxel and carboplatin failed to prolong OS versus chemotherapy alone. However, there was a trend towards longer OS with atezolizumab plus nab-paclitaxel and carboplatin in the PD-L1-high subgroup (7). More recently, the phase III IMpower 110 trial showed that atezolizumab monotherapy outperforms chemotherapy for NSCLC patients with high PD-L1 expression, irrespective of histologic type (8). Therefore, both atezolizumab monotherapy or atezolizumab plus chemotherapy could be treatment options for patients with advanced NSCLC and high PD-L1 expression, defined as either greater than 50% PD-L1 expression in the tumor cells (TC) or greater than 10% PD-L1 expression in immune cells (IC) under SP142 PD-L1 immunohistochemistry (IHC) assay (PD-L1 TC3/IC3). Currently, whether the subtraction of chemotherapy from atezolizumab plus chemotherapy could be non-inferior is controversial owing to the lack of head-to-head comparisons.

As such, we estimated the efficacy and safety of atezolizumab plus chemotherapy versus atezolizumab alone for the first-line treatment of advanced NSCLC patients in a PD-L1 TC3/IC3 subgroup through an indirect comparison meta-analysis.

## Methods

### Study Eligibility

This meta-analysis was prepared and written following the Preferred Reporting Items for Systematic Reviews and Meta-Analyses (PRISMA) statement. From the PubMed, EMBASE, and Cochrane Central controlled trial registries, we identified qualified randomized controlled trials that compared atezolizumab plus chemotherapy or atezolizumab alone with chemotherapy for first-line treatment of advanced NSCLC patients. We searched for studies using keywords including atezolizumab, PD-1, PD-L1, non-small cell lung cancer, and randomized controlled trial (**Supplemental Methods**). We also searched abstracts from major conferences of the American Society of Clinical Oncology (ASCO), the European Society of Medical Oncology (ESMO), the American Association for Cancer Research (AACR), and the World Congress on Lung Cancer (WCLC). These clinical studies were limited to those published in English before October 1, 2020.

### Data Extraction

Data were extracted with a pre-determined information table. The primary results of this study included PFS, OS, ORR, and TRAEs. We derived the hazard ratios (HRs) and 95% confidence intervals (CIs) for OS and PFS, and the dichotomous data for ORR and TRAEs. Other items included the design of the trial, the number of patients registered, the year of publication or conference presentation, the median follow-up time, the clinical pathological characteristics of the patients, including histology, ECOG score, smoking status, and PD-L1 expression.

### Data Analyses

Direct comparisons were conducted between arm A (atezolizumab plus chemotherapy) against arm B (chemotherapy), and arm C (atezolizumab) against arm B (chemotherapy). The pooled measurement of PFS and OS were shown with HRs, 95% Cis, and *P* values calculated by the inverse-variance-weighted method, while the estimation of ORR and TRAEs were pooled with the relative risks (RRs), 95% Cis, and *P* values *via* the Mantel Haenszel method. Fixed-effect or random-effect models were based on the heterogeneity between studies.

Indirect comparison between arm A and arm C was bridged by arm B. The adjusted indirect comparison was calculated by the following formula (9): log HR_AB_ = log HR_AC_-log HR_BC_. The standard error (SE) for the log HR was SE (*logHR*AB) = $\sqrt{SE{\left(logHRAC\right)}^{2}+SE{\left(logHRBC\right)}^{2}}$. RR was calculated similarly to the above method. HR < 1 or RR > 1 demonstrates that atezolizumab plus chemotherapy is superior to atezolizumab alone, and vice versa. All statistical analyses were performed using STATA (version 12.0). Statistical significance was defined as a two-sided P < 0.05.

## Results

**Figure 1:**
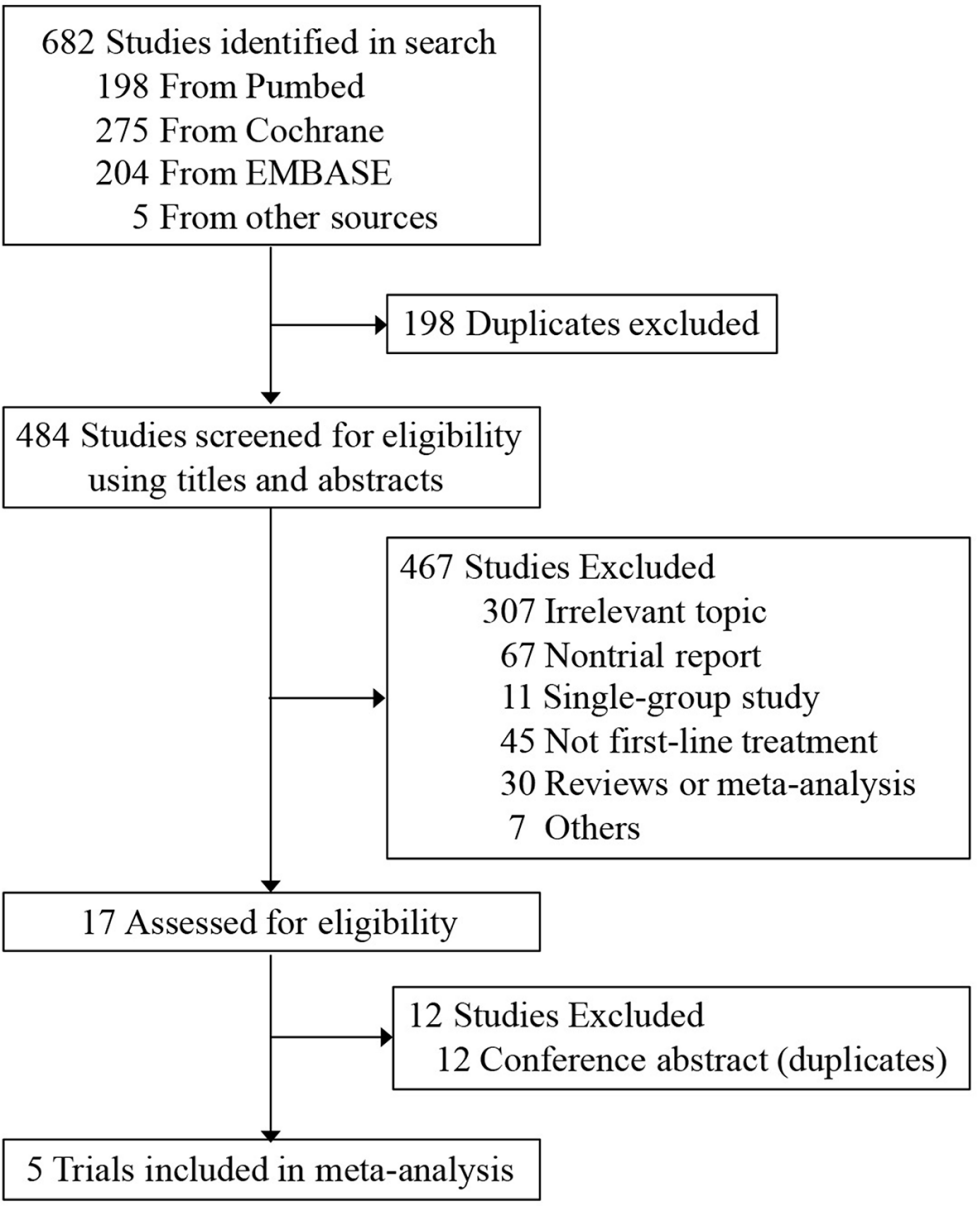
PRISMA flow diagram of the meta-analysis.

The PRISMA flow diagram of our meta-analysis was shown in **Figure 1**. The quality assessment of risk of bias was presented in **Supplemental Table 1**. In total, five trials involving 616 patients fulfilled the predefined inclusion criteria. The principal characteristics and outcomes of the included trials were summarized in **Table 1**. In all clinical trials, patient characteristics between the experimental and control groups were well balanced. Patients with non-squamous NSCLC were recruited in three trials, one for squamous NSCLC and the other one for both histologies. Four trials compared atezolizumab plus chemotherapy versus chemotherapy and one trial compared atezolizumab alone versus chemotherapy. Additionally, IMpower130 study was a three-arm randomized trial that investigated atezolizumab with or without bevacizumab, an inhibition of vascular endothelial growth factor, plus chemotherapy versus chemotherapy alone. According to practice guidelines, all five trials were treated with standard-of-care chemotherapy regimens. The median follow-up time ranged from 15.7 to 28.4 months. PFS, OS, and AE information were provided for all five trials; however, ORR data was not reported in IMpower 130. The PD-L1 expression on tumor cells and tumor-infiltrating immune cells were measured by the SP142 IHC assay (Ventana Medical Systems). The safety summary was presented detailed in **Table 2**.

**Table 1:** Baseline characteristics and available endpoints of PD-L1 TC3/IC3 WT patients in included trials.

Baseline Characteristics	IMpower110		IMpower130		IMpower131		IMpower132		IMpower150	
	Atezo	Chemo	Atezo+ Chemo	Chemo	Atezo+ Chemo	Chemo	Atezo+ Chemo	Chemo	Atezo+ Chemo	Chemo
**All eligible patients**	277	277	451	228	343	340	292	286	359	337
**Median age-years**	64	65	64	65	65	65	64	63	63	63
**Male sex (%)**	70.8	69.7	59	59	81.6	81.5	65.8	67.1	60.0	59.8
**ECOG^a^ score (%)**										
**0**	35.0	36.8	42	40	33.5	32.4	43.2	40.1	40.1	45.1
**1**	65.0	63.2	58	60	66.2	67.4	NE	NE	59.9	54.9
**Smoking status (%)**										
**Current/former**	86.6	87.3	89	92	90.7	92.9	87.3	89.5	79.5	80.8
**Never**	13.4	12.6	11	7	9.3	6.8	12.7	10.5	20.5	19.2
**Unknown**	0	0	0	1	0	0.3	0	0	0	0
**Histologic type (%)**										
**Squamous**	30.7	30.3	0	0	100	100	0	0	0	0
**Non-squamous**	69.3	69.7	100	100	0	0	100	100	99.3	98.4
**PD-L1 TC3/IC3 WT (%)**	38.6	35.4	20.0	18.0	13.7	12.9	14.2	11.9	19.8	19.3
**Endpoints**	OS		PFS and OS		PFS and OS		PFS and OS		PFS and OS	
**Interventions**	Atezo^b^	Nsq: AP^c^ Sq: GP^c^	Atezo+TC^d^	TC	Atezo+CP/CnP^e^	CnP	Atezo+TC/TP^f^	TC/TP	Atezo+Bev+CP	Bev+CP^g^
**Follow-up time (m)**	15.7		19.2		26.8		28.4		19.7	
**OS (m), HR (95%CI)**	20.2 vs. 13.1 0.59 (0.40, 0.89)		17.3 vs. 16.9 0.84 (0.51, 1.39)		23.4 vs. 10.2 0.48 (0.29, 0.81)		NE vs. 26.9 0.73 (0.31, 1.73)		25.2 vs. 13.2 0.67 (0.42–1.06)	
**PFS (m), HR (95%CI)**	8.1 vs. 5.0 0.63 (0.45, 0.88)		6.4 vs. 4.6 0.51 (0.34, 0.77)		10.1 vs. 5.1 0.41 (0.25, 0.68)		10.8 vs. 6.5 0.46 (0.22, 0.96)		15.4 vs. 6.9 0.33 (0·22–0·51)	
**ORR (%)**	38.3 vs. 28.6		NE		61.7 vs. 31.8		72.0 vs. 55.0		68.9 vs. 49.3	

^a^Performance-status evaluation of the Eastern Cooperative Oncology Group.

^b^Atezolizumab (1200 mg intravenously).

^c^AP: pemetrexed (500 mg/m^2^ Q3W) + cisplatin (75 mg/m^2^ Q3W) /carboplatin (AUC=6 Q3W); GP: gemcitabine (1250/m^2^) + cisplatin (75mg/m^2^) or gemcitabine (1000 mg/m^2^) + carboplatin (AUC=5 Q3W).

^d^Atezo+TC: Atezolizumab (1200 mg intravenously) + carboplatin (6 mg/mL/min Q3W) + nab-paclitaxel (100 mg/m2, every week).

^e^Atezo+CP: Atezolizumab (1200 mg intravenously) + carboplatin (6 mg/mL/min Q3W) + pemetrexed (200 mg/m^2^ Q3W, 175 mg/m2 for Asian race), CnP : nab-paclitaxel (100 mg/m2, every week).

^f^Atezo+TC/TP: Atezolizumab (1200 mg intravenously) + carboplatin (6 mg/mL/min Q3W) or cisplatin (75mg/m^2^) + pemetrexed (500 mg/m^2^ Q3W).

^g^Bev+CP: bevacizumab (15mg/kg Q3W) + carboplatin (6 mg/mL/min Q3W)+ paclitaxel (200mg/m² Q3W, 175mg/m² for Asian patients) PD-L1, programmed cell death-ligand 1; TC, tumor cell; IC, immune cell; ECOG, Eastern Cooperative Oncology Group; OS, overall survival; PFS, progression-free survival; ORR, objective response rate; mDOR, median duration of response; 95%CI, 95% confidence interval (CI); m, months.

**Table 2:** Safety summary.

	IMpower110		IMpower130		IMpower131		IMpower132		IMpower150	
	Atezo	Chemo	Atezo+Chemo	Chemo	Atezo+Chemo	Chemo	Atezo+Chemo	Chemo	Atezo+Chemo	Chemo
**All cause AEs, n (%)**	258 (90.2)	249 (94.7)	471 (99.6)	230 (99.1)	332 (99.4)	324 (97.0)	287 (98.6)	266 (97.1)	386 (98.2)	390 (99.0)
**Grade 3–5 AEs, n (%)**	97 (33.9)	149 (56.7)	406 (85.8)	177 (76.3)	277 (83.0)	235 (70.4)	208 (71.5)	166 (60.0)	274 (69.7)	251 (63.7)
**AE leading to any treatment withdrawal, n (%)**	18 (6.3)	43 (16.3)	125 (26.4)	51 (22.0)	102 (30.5)	58 (17.4)	83 (28.5)	50 (18.2)	133 (33.8)	98 (24.9)
**AE related death, n (%)**	2 (0.7)	3 (1.1)	8 (1.7)	1 (0.4)	4 (1.2)	3 (0.9)	11 (3.8)	8 (2.9)	11 (2.8)	9 (2.3)

Atezo, atezolizumab; Chemo, chemotherapy; AEs, adverse effects.

### Direct Meta-Analysis

**Figure 2:**
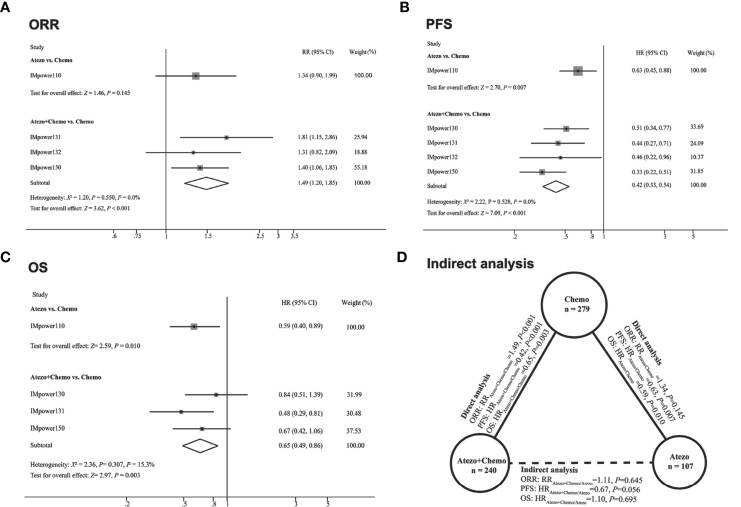
Direct comparisons and indirect comparisons of efficacy between Atezolizumab (Atezo) plus chemotherapy versus Atezolizumab for patients with high PD-L1 expression. **(A–C)** showed the forest plot of RR and HR directly comparing ORR **(A)**, PFS **(B)**, and OS **(C)** between Atezo plus chemotherapy or Atezo alone. The size (square) of the data marker corresponds to the weight of the study in the meta-analysis. The horizontal line across the square represents 95% confidence interval (CI). Based on the meta-analysis, the diamond represents the overall effect of the estimation. **(D)** showed the results of indirect analysis for ORR, PFS, and OS between Atezo plus chemotherapy and Atezo. Solid lines represented the existence of direct comparisons between treatment regimens, while the dotted line represented the indirect comparison between Atezo plus chemotherapy versus Atezo. All statistical tests were two-sided.

Significant difference of ORR was observed in favor of atezolizumab plus chemotherapy versus chemotherapy (RR_atezo + chemo/chemo_ 1.49, 95% CI 1.20–1.85; Z = 3.62, *P＜*0.001). And in terms of atezolizumab versus chemotherapy, the pooled RR was 1.33 (RR_atezo + chemo/chemo_ 1.34, 95% CI 0.90–1.99; Z = 1.46, *P* = 0.145) (**Figure 2A**). For PFS, atezolizumab plus chemotherapy significantly reduced the risk of disease progression or death compared with chemotherapy (HR_atezo + chemo/chemo_, 0.42; 95% CI 0.33–0.54; Z = 7.09, *P* = 0.001). In addition, atezolizumab monotherapy was also correlated with longer PFS versus chemotherapy (HR_atezo/chemo_ = 0.63; 95% CI, 0.45–0.88; Z = 2.70, *P* = 0.007) (**Figure 2B**). In terms of OS, compared with chemotherapy, both atezolizumab plus chemotherapy (HR_atezo+ chemo/chemo_ = 0.65; 95% CI, 0.49–0.86; Z = 2.97, *P* = 0.003) and atezolizumab monotherapy (HR_atezo/chemo_ = 0.59; 95% CI, 0.40–0.89; Z = 2.59, *P* = 0.010) significantly reduced the risk of death (**Figure 2C**).

### Indirect Meta-Analysis

The correlation of indirect analysis was shown in **Figure 2D**. The results showed that the efficacy of the atezolizumab combined chemotherapy group was not superior to that of the atezolizumab monotherapy including ORR (RR_atezo + chemo/atezo_ 1.11, *P* = 0.645) and OS (HR_atezo+ chemo/atezo_ 1.10, *P* = 0.695). In addition, atezolizumab combined with chemotherapy did not show a significant difference in PFS compared with atezolizumab monotherapy in terms of PFS (HR_atezo + chemo/atezo_ = 0.67, *P* = 0.056).

**Figure 3:**
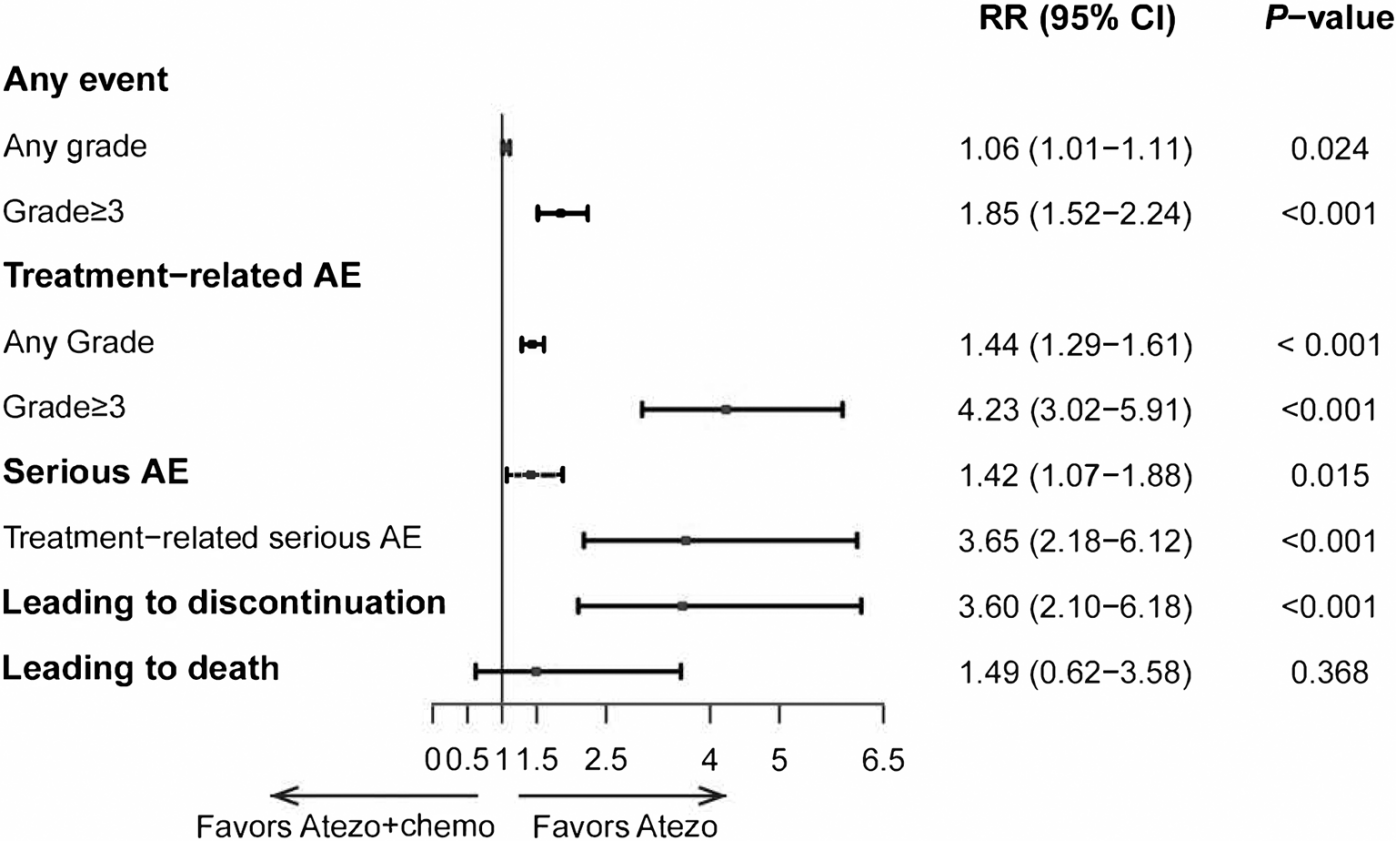
Indirect comparisons of safety between Atezolizumab (Atezo) plus chemotherapy versus Atezolizumab for patients. The forest plot showed RRs for TRAEs between Atezo plus chemotherapy versus Atezo alone. The horizontal line crossing the square represents the 95% confidence interval (CI). The diamonds represent the estimated overall effect, based on the meta-analysis.

The rate of all grades (RR, 1.06; 95% CI, 1.29-1.61) and ≥ grade 3 (RR, 4.23; 95% CI, 3.02-5.91) TRAEs were both significantly higher in atezolizumab plus chemotherapy than those in the atezolizumab monotherapy group (**Figure 3**). Additionally, the rate of TRAEs leading to drug discontinuation occurred more frequently in those receiving atezolizumab plus chemotherapy than in those treated with atezolizumab monotherapy (RR, 3.60; 95% CI, 2.10-6.18). Treatment-related deaths were similar between atezolizumab alone and atezolizumab with chemotherapy groups (RR, 1.49; 95% CI, 0.62-3.58).

## Discussion

To the best of our knowledge, this is the first study to compare the efficacy and safety of atezolizumab plus chemotherapy and atezolizumab monotherapy in advanced NSCLC patients with high PD-L1 expression through indirect analysis. This hypothesis-generating meta-analysis showed that atezolizumab monotherapy was non-inferior to atezolizumab plus chemotherapy as a first-line treatment for patients with advanced NSCLC and PD-L1 TC3/IC3 expression. Unsurprisingly, patients receiving atezolizumab monotherapy experienced fewer TRAEs than those receiving combination therapy.

Currently, atezolizumab in combination with carboplatin plus nab-paclitaxel or with bevacizumab, carboplatin, and paclitaxel have been officially approved as a first-line treatment for metastatic non-squamous NSCLC lacking EGFR mutation or ALK rearrangements, based on the OS benefit over their comparator chemotherapy alone (5, 6). For squamous cell lung cancer, atezolizumab plus chemotherapy was significantly associated with prolonged progression-free survival, though this was not translated into overall survival benefit. Yet, there was a tendency towards improved OS with the atezolizumab plus chemotherapy group for patients with high PD-L1 expression (HR, 0.56; 95% CI, 0.32-0.99) (7). These results indicate the atezolizumab plus chemotherapy might yield survival benefit for patients with advanced NSCLC and high PD-L1. We confirmed this observation by conducting a direct meta-analysis of randomized controlled trials. The results showed that atezolizumab plus chemotherapy outperforms chemotherapy in terms of ORR (RR, 1.49; 95% CI, 1.20-1.85), PFS (HR, 0.42; 95% CI, 0.33-0.54), and OS (HR, 0.65; 95% CI, 0.49-0.86). Theoretically, combining immune checkpoint inhibitors with chemotherapy could achieve additive anti-tumor activity compared with ICIs alone: chemotherapy enhances the recognition and elimination of tumor cells by the host immune system; moreover, chemotherapy might optimize the tumor immune microenvironment (10–13). However, these combinatory strategies are challenged by their higher risks of TRAEs, discontinuation rates, and economic cost (14, 15). It is of important clinical significance to explore biomarkers to select the subgroup of NSCLC patients benefiting from chemotherapy-free treatment options. Among them, PD-L1 expression was the most extensively studied biomarker (2, 16, 17). A previous study found that, for patients whose tumoral PD-L1 expression stained by 22C3 clone was 50% or more, pembrolizumab plus chemotherapy produced better ORR and PFS than pembrolizumab alone. However, the OS was not different between pembrolizumab plus chemotherapy and pembrolizumab alone (3). This implies that the upfront application of chemotherapy for the combination with immunotherapy could lead to short-term clinical benefit but not OS benefit. For atezolizumab, the phase II clinical trial BIRCH has also demonstrated atezolizumab monotherapy was associated with good tolerability and efficacy in patients with PD-L1-selected advanced NSCLC across lines of therapy, especially in PD-L1 TC3/IC3 patients (16). Based on the promising results of BIRCH, the randomized controlled phase III IMpower110 study were conducted to evaluate the efficacy and safety of atezolizumab monotherapy as the first-line treatment in PD-L1 selected patients. Encouragingly, the final OS analysis of IMpower110 indicated atezolizumab monotherapy showed statistically significant and clinically meaningful OS, PFS, ORR, and duration of response (DOR) improvement in the TC3/IC3 population compared with platinum-based chemotherapy (4). Therefore, single-agent atezolizumab has been approved as one of the preferred first-line regimens for patients with metastatic NSCLC and a high PD-L1 expression. Considering these results, it is difficult to decide whether patients with high PD-L1 could benefit more from atezolizumab in combination with chemotherapy or atezolizumab alone. In light of the lack of such head-to-head comparisons and the urgent need for this evidence to guide clinical practice, we performed this indirect comparison for atezolizumab plus chemotherapy and atezolizumab as a single agent.

Our pooled analysis shows that the addition of chemotherapy to atezolizumab was not beneficial compared with atezolizumab alone in terms of OS (HR 1.10, *P* = 0.695). Additionally, the outcomes of ORR and PFS seem favorable in the combined treatment group, but the difference was not significant (ORR: RR, 1.11, *P* = 0.645; PFS: HR, 0.67, *P* = 0.056). However, the rate of all grades (RR, 1.06; 95% CI, 1.29-1.61) and ≥ grade 3 (RR, 4.23; 95% CI, 3.02-5.91) TRAEs were both obviously higher in the combination group. These results further provide support for the recommendation from NCCN that patients with advanced NSCLC and high PD-L1 could be treated preferentially by atezolizumab monotherapy rather than in combination with chemotherapy.

Our results support the predictive role of PD-L1 TC3/IC3 in the selection of advanced NSCLC patients who would benefit from single-agent atezolizumab to some extent. The underlying explanation of the lack of addictive benefit from the combination of chemotherapy to atezolizumab may include the following. Firstly, the subgroup of patients with PD-L1 TC3/IC3 possesses a specific tumor microenvironment where the tumor cells themselves and the surrounding immune cells collectively suppressed CD8+ cytotoxic T lymphocytes (CTLs)-mediated immune surveillance and attacked their high PD-L1 expression. Therefore, by removing the interaction between PD-1 and PD-L1, atezolizumab could induce anti-tumor immunity to a greater extent. Besides, it is likely that chemotherapy could not trigger further anti-tumor immunity in PD-L1 TC3/IC3 patients because they are a “hot tumor” by nature.

The advantage of our work lies on the quality of the evidence extracted and applied in the meta-analysis. The origin data were obtained from five prospective randomized controlled trials involving more than 2000 patients. The antibody of immunotherapy and method of detecting PD-L1 expression are the same. Therefore, the meta-analysis could obviously reduce the heterogeneity between studies by collecting data, thereby overcoming the problem of insufficient strength of multiple experiments, which makes indirect analysis feasible to a certain extent. In addition, several limitations of the study should also be considered. Firstly, our meta-analysis is based on study-level data, but not individual patients’ data. The analyses could not be adjusted for patients’ characteristics. Secondly, due to the absence of a head-to-head comparison between atezolizumab and atezolizumab plus chemotherapy, such a comparison could only be made *via* an indirect meta-analysis. Such an approach will face methodological challenges. However, we believe the quality of the included trials and the similarity between the comparative populations together make the indirect comparison more convincing (9). Finally, the data extracted in our meta-analysis are from subgroup analyses which means the sample is not large enough. Thus, the interpretation of our results requires extra caution. In view of these limitations, a randomized head-to-head trial will be urgently needed to directly compare the efficacy and safety between atezolizumab plus chemotherapy and atezolizumab alone.

Limitations aside, this study for the first time compared the efficacy and safety of atezolizumab plus chemotherapy with atezolizumab alone for advanced NSCLC. Atezolizumab monotherapy might be a preferred first-line treatment option for patients with advanced NSCLC and TC3/IC3 PD-L1 expression.

## Data Availability Statement

The raw data supporting the conclusions of this article will be made available by the authors, without undue reservation.

## Author Contributions

D-NL, W-QL, and B-WY contributed to data acquisition, data interpretation, statistical analysis, and drafting of the manuscript. Y-PL and X-JQ contributed to the study design, data acquisition, data interpretation, and statistical analysis. All authors contributed to the article and approved the submitted version.

## Funding

This work was supported by grants from the National Natural Science Foundation of China under Grant (No.82003296), The Key Research and Development Program of Liaoning Province under Grant (2018225060), and the Technological Special Project of Liaoning Province of China under Grant (2019020176-JH1/103).

## Conflict of Interest

The authors declare that the research was conducted in the absence of any commercial or financial relationships that could be construed as a potential conflict of interest.

## Abbreviations

NSCLC, Non-small-cell lung carcinoma; PD-1, Programmed cell death 1; PD-L1, Programmed cell death-ligand 1; RCT, randomized controlled trials; PRISMA, Preferred Reporting Items for Systematic Reviews and Meta-Analyses; AACR, American Association for Cancer Research; ESMO, European Society of Medical Oncology; ASCO, American Society of Clinical Oncology; WCLC, World Conference on Lung Cancer; EMA, European Medicines Agency; FDA, drug administration; AEs, Adverse events; TRAEs, Treatment related adverse effects; CI, Confidence interval; HRs, Hazard ratios; RR, Relative risk; TC, tumor cells; IC, immune cells; DOR, Duration of response; ORR, Objective response rate; OS, Overall survival; PFS, Progression-free survival; ECOG, Eastern Cooperative Oncology Group; Immunohistochemistry, IHC.
