# Supplementary material for: Atezolizumab Monotherapy or Plus Chemotherapy in First-Line Treatment for Advanced Non-Small Cell Lung Cancer Patients: A Meta-Analysis
Source: Front Immunol. 2021 Jun 2;12:666909. doi: 10.3389/fimmu.2021.666909 (PMC8212861; doi:10.3389/fimmu.2021.666909)
Supplement: Supplementary file 1 [file DataSheet_1.doc]

**Supplemental Methods**

**Search strategies and number of studies achieved from PubMed, EMBASE, and Cochrane database**

**Pubmed: 198 Results**

(((("Carcinoma, Non-Small-Cell Lung"[Mesh]) OR ((((NSCLC[Title/Abstract] OR "Non Small Cell"[Title/Abstract] OR "Non-Small-Cell"[Title/Abstract] OR "Non- Small Cell"[Title/Abstract] OR "Non-Small Cell"[Title/Abstract]))) AND lung[Title/Abstract]))) AND (("atezolizumab" OR "Tecentriq"[Title/Abstract] )) AND ((("clinical trials as topic"[MeSH Terms] OR "trial"[Title/Abstract] OR "study"[Title/Abstract])))

**EMBASE: 204 Results**

(‘atezolizumab’/exp OR ‘Tecentriq’:ab,ti) AND (('non-small cell lung cancer'/exp OR ('lung':ab, ti AND (‘NSCLC’:ab, ti OR ‘Non-Small Cell’:ab,ti OR ‘Non-Small-Cell’:ab, ti OR ‘Non-Small Cell’:ab, ti OR ‘Non-Small Cell’:ab,ti))) AND (‘randomized controlled trial’/exp))

**Cochrane: 275 results, 267 trials**

#1 MeSH descriptor: [Carcinoma, Non-Small-Cell Lung] explode all trees

#2 “lung” AND ("Non-Small Cell" OR "Non-Small Cell" OR "Non-Small-Cell") OR "NSCLC"

#3 #1 OR #2

#4 MeSH descriptor: [Atezolizumab] explode all trees

#5" Atezolizumab" OR " anti-PDL1" OR "Tecentriq" OR "MPDL3280A" OR "MPDL-3280A" OR "RG7446" OR " RG-7446"

#6 #4 OR #5

#7 MeSH descriptor: [Randomized Controlled Trial] explode all trees

#8 "Randomized Controlled Trial" OR "Controlled Clinical Trials, Randomized" OR "Clinical Trials, Randomized" OR "Trials, Randomized Clinical" OR "Clinical trial" OR "Clinical trials

#9 #7 OR #8

#10 #3 AND #6 AND #9

**Supplemental Table 1**. Quality assessment: risk of bias by Cochrane Collaboration’s tool.

| **Trial** | **Sequence generation** | **Allocation**  **concealment** | **Blinding** | **Incomplete outcome data** | **Selective reporting** |
| --- | --- | --- | --- | --- | --- |
| IMpower110 | Adequate | Adequate (Central  allocation) | Adequatea | Adequate | Adequate |
| IMpower130 | Adequate | Adequate (Central  allocation) | Adequatea | Inadequate datab | Adequate |
| IMpower131 | Adequate | Adequate (Central  allocation) | Adequatea | Adequate | Adequate |
| IMpower132 | Adequate | Adequate (Central  allocation) | Adequatea | Adequate | Adequate |
| IMpower150 | Adequate | Adequate (Central  allocation) | Adequatea | Adequate | Adequate |

a The sponsor, investigator and subject were aware of the treatment administration but the response to treatment was assessed by means of blinded, independent, central radiologic review. ^b^ Absence of objective response rate (ORR)
